# Mode of Distinguishing between Nervous Idiopathic Albuminuria and the Albuminuria of Diseased Kidneys

**Published:** 1866-08

**Authors:** 


*Mode of distinguishing between Nervous Idiopathic Albuminuria and the Albuminuria of Diseased Kidneys.*—The principle upon which M. Corlieu founds the test by which he distinguishes between these two forms is, that when the kidneys are healthy the urine possesses the smell of the odorous substances introduced into the system. He says that if such substances as cubebs, turpentine, etc., be ingested, they will give their characteristic odor to the urine in cases of albuminuria, provided the kidneys be healthy; but if the kidneys be diseased, the odor of these substances can not be detected, even though they have been previously introduced into the system.—*N. Y. Medical Journal.*

